# Correspondence

**Published:** 1865-12

**Authors:** 


### Correspondence.

CINCINNATI, December 15, 1865.

To the Editor of the Buffalo Medical and Surgical Journal:

I am very favorably impressed with Dr. Colterson's remarks on Military Surgery in the main, but in regard to the treatment of hospital gangrene, I desire to say a few words.

My own experience was that the first and most important thing to be attended to was a full support of the vital forces by stimulants and good nourishment, and when erysipelas or gangrene began to make its appearance, it was often arrested by the free use of tinct. ferri chlorid, used internally, either alone or combined with quinine, when the patient was emaciated, tongue dry, and tending to a typhoid condition. The quinine combined with the iron seemed to have a most salutary influence in arresting the threatening trouble. In many of the hospitals, and in fact nearly all of which I could learn in this department, hospital gangrene did not make its appearance to any extent until after the second year, which would seem to indicate that the cause was cumulative, or the result of continuing for sometime in one place, and the fact that changing the patients to new wards or tents out of doors with decided advantage, which was often done, would go far to prove that the subtle influence accumulated in wards long used for wounds, in spite of the most careful attention to cleanliness. The cases of hospital gangrene, of a formidable character, first made their appeaeance at the Washington Park Hospital, in this city, amongst the wounded from the battle of Stone River, in January, 1863, and we were seldom without them until the close of the hospital in May, 1865.

In the local treatment I used at different times the various popular caustics, but they often failed. The bromine did not give us the satisfaction promised, although carefully applied under the direction of its champion, Dr. Goldsmith. The nitric acid also often failed to arrest the disease, but the actual cantery in the most severe cases always arrested the trouble after the third or fourth application, and with much less suffering to the patient. It was our practice always to put the patient under the influence of chloroform, and apply the cautery freely to the edges of the wound as the diseased action seemed to confine itself to the adipose tissue, and then apply an anodyne poultice until the diseased parts sloughed off, after which it was treated as a simple ulcer. I regard the actual cautery as by far the most valuable escharotic and perfectly safe in the hands of the judicious surgeon, of anything now within our knowledge, and since the days of chloroform, like the knife, it is divested of much of its formidable character.

Yours, O. D. Horton.

---

Racine, Wisconsin, Dec. 12, 1865.

Mr. Editor:—In the December, 1852, number of the *Buffalo Medical Journal*, is an article upon Cholera, in which I suggested the external application of cold water, as an adjuvant in the remedial management of that disease.

During the prevalence of the disease in Chicago in 1854, I had ample opportunity to fully test the efficacy of the practice, which more than realized my expectations. In numerous cases, even in collapse, when the other usual means had failed, I succeeded in bringing about re action and saving the patient.

My method was as follows:—The patient being divested of all clothing, except the chimise, in the case of females, was placed upon the floor, or in a bathing tub, the head and shoulders elevated, and thirty or forty gallons of *cold* water *dashed forcibly* (half a pail at a time) over the head and shoulders, down the spine, and upon the chest. The patient was then immediately placed between blankets and rubbed gently with dry mustard. The following case will illustrate:

Mrs. L——, the wife of a well-known citizen of Chicago, was under the care of the late Dr. Cheeny, who, finding her sinking in spite of best efforts, consulted me. Her condition was apparently hopeless, she had frequent involuntary characteristic dejections from the bowels; her stomach rejected everything taken into it; there was intolerable thirst and violent spasm of the voluntary muscles. Her respiration was labored; pulse scarcely perceptible at the wrist; the surface and extremities cold; the eyes and features shrunken. She was ordered an enema of tannin and lead in cold water, and immediately subjected to the cold affusion followed by friction with the mustard, whereby warmth was restored to the surface and extremities; the vomiting and cramping ceased; there was no dejection for several hours; the respiration became free, pulse was fuller and more distinct, the thirst was entirely relieved, and from that moment she convalesced.

I could cite many other similar cases with like result, to which many physicians and other citizens of Chicago will bear testimony. But my object is only to call the attention of the profession to the remedial measure.

In this connection I will state that I have found no internal remedy more efficacious in controlling serous diarrhœa, especially that preceding, or rather initiatory of cholera than the extract of *nux vomica*, I have usually prescribed it in combination, thus:

℞ Ext. nucis vomica, alch. gr. iv.
Pil hydrargyri vel.
Hydrarg. chlo. mite gr. viii.
Di-sulph. quinia gr. x.
Opii pulv. gr. ij, c. iv.
Camphora pulv.
Capsici pulv. aa. gr. iv.
M. ft. pil. viii.
Sig. one or two every four hours.

My brother, the late Dr. E. A. C. Page, who was, in 1849, connected with one of the New York City Dispensaries, and saw a good deal of cholera in that city, suggested the annexed formula:

℞ Spts. ammon, arom.
Ether sulph.
Tinct. kino.
Tinct. capsici, aa. ℥ ss.
Tinct. opii ʒ iij, c. iv.
Spts. mentha pip. ℥ i.
Aqua camphora ℥ ij ss.
Syr. rhei arom. ℥ ij.
Signa. M.

For *diarrhœa* one or two teaspoonsful in water, every three or four hours; for *cholera* a table-spoonful every half hour.

So efficient has it proved that it is prepared and vended as Dr. Page's Cholera and Diarrhœa Elixir, by our druggists, and may be found in almost every family in this vicinity. My own experience in its use warrants me in recommending it as a proper medicine to be kept by families, to be used in the incipiency of an attack of cholera until a physician can be consulted.

Yours, JOHN L. PAGE.

